# N-Terminal Domain of Feline Calicivirus (FCV) Proteinase-Polymerase Contributes to the Inhibition of Host Cell Transcription

**DOI:** 10.3390/v8070199

**Published:** 2016-07-20

**Authors:** Hongxia Wu, Shaopo Zu, Xue Sun, Yongxiang Liu, Jin Tian, Liandong Qu

**Affiliations:** 1 Division of Zoonosis of Natural Foci, State Key Laboratory of Veterinary Biotechnology, Harbin Veterinary Research Institute, Chinese Academy of Agricultural Sciences, 427 Maduan Street, Nangang District, Harbin 150001, China; whx450650@163.com (H.W.); 18745026359@163.com (S.Z.); shellyfree@126.com (X.S.); liuyongxiang0726@foxmail.com (Y.L.); 2 College of Veterinary Medicine, Northeast Agricultural University, Harbin 150001, China

**Keywords:** FCV, PP, inhibition, key function sites, transcription

## Abstract

Feline Calicivirus (FCV) infection results in the inhibition of host protein synthesis, known as “shut-off”. However, the precise mechanism of shut-off remains unknown. Here, we found that the FCV strain 2280 proteinase-polymerase (PP) protein can suppress luciferase reporter gene expression driven by endogenous and exogenous promoters. Furthermore, we found that the N-terminal 263 aa of PP (PP_N-263_) determined its shut-off activity using the expression of truncated proteins. However, the same domain of the FCV strain F9 PP protein failed to inhibit gene expression. A comparison between strains 2280 and F9 indicated that Val27, Ala96 and Ala98 were key sites for the inhibition of host gene expression by strain 2280 PP_N-263_, and PP_N-263_ exhibited the ability to shut off host gene expression as long as it contained any two of the three amino acids. Because the N-terminus of the PP protein is required for its proteinase and shut-off activities, we investigated the ability of norovirus 3C-like proteins (3CLP) from the GII.4-1987 and -2012 isolates to interfere with host gene expression. The results showed that 3CLP from both isolates was able to shut off host gene expression, but 3CLP from GII.4-2012 had a stronger inhibitory activity than that from GII.4-1987. Finally, we found that 2280 PP and 3CLP significantly repressed reporter gene transcription but did not affect mRNA translation. Our results provide new insight into the mechanism of the FCV-mediated inhibition of host gene expression.

## 1. Introduction

*Caliciviridae* are positive-stranded RNA viruses containing four recognized genera: *Norovirus*, *Sapovirus*, *Lagovirus* and *Vesivirus*. Norovirus is the most common pathogen that causes human viral gastrointestinal disease worldwide. Most caliciviruses cannot be successfully cultivated in vitro, which severely restricts the further study of these viruses. However, murine norovirus (MNV) (genus *Norovirus*) and feline calicivirus (FCV) (genus *Vesivirus*) have been widely used as models to analyze the characteristics of the calicivirus life cycle due to their culturability [1]. FCV infection affects the oral cavity and upper respiratory tract and is a good model with many advantages, such as a mature reverse genetics system [2] and virus isolation, and culture technology.

FCV possesses a linear, positive-sense, single-stranded RNA genome of 7.7 kb. The VPg protein is covalently linked to the 5′-end of both the genomic and subgenomic RNAs [3]. VPg acts as a cap substitute to recruit ribosomes and initiate translation [4,5]. After FCV infection, ORF1 is translated using the host translation system and produces a large polyprotein that undergoes cotranslational proteolytic processing by itself. Finally, it is cut into five nonstructural proteins, p5.6, p32, p39, p30, VPg, Proteinase-polymerase (PP) [6]. PP and its precursor have cysteine proteinase activity and are responsible for the proteolytic processing [7]. When viral protein synthesis reaches a certain level, translation is inhibited and viral genome replication begins [8]. The FCV PP protein is the only RNA-dependent RNA polymerase (RdRp) encoded by the virus and is also required for genomic replication [9]. FCV RNA synthesis occurs in the cytoplasm in a perinuclear region. When RNA synthesis starts, nucleolin relocalizes from the nucleoli to the nucleoplasm as well as to the perinuclear area, where it colocalizes with PP [10].

RNA viruses have evolved a variety of strategies to use host resources for their replication. About half of host protein synthesis was repressed within 5 h after FCV infection [11]. However, virus capsid protein expression peaks at 6 h post-infection (hpi), and the host cells suffer from apoptosis at 9 hpi [11]. A previous study reported that the translation initiation factors eIF4GI and eIF4GII were cleaved upon FCV infection [11], which did not depend on the caspase-3-mediated apoptosis pathway due to cleavage at specific sites [12]. Because the L protease of foot-and-mouth virus [13] and the 2A protease of poliovirus [14] can directly induce the cleavage of targets, it is logical that as the only protease of FCV, PP may have a similar function; however, the role of PP in the process of FCV infection-mediated cleavage of host proteins is unknown. A further study showed that the 3C-like protein of human norovirus (strain MD145-12) and PP of FCV (strain Urbana) were demonstrated to cleave host poly(A)-binding protein (PABP) and hinder the recycling of ribosomes during translation, which is a potential strategy that *Caliciviridae* use to inhibit host cell protein translation [15]. Both eIF4GI and eIF4GII serve as scaffolds to bridge the mRNA and the ribosome. However, the PP protein has both protease and polymerase activity, and it is unknown which functional region or key sites are required for the inhibition of host gene expression and whether the PP protein affects host gene expression by suppressing gene translation.

In this study, we investigated how FCV strain 2280 PP affects host gene expression driven by different promoters, and we identified the key domain and sites that are required for inhibiting host gene expression. Moreover, we found that the 2280 PP protein affects host gene expression by suppressing gene transcription. This study is helpful to further understand the function of the FCV PP protein.

## 2. Materials and Methods

### 2.1. Cells and Virus

Crandell-Reese feline kidney (CRFK) cells and BHK-21 cell lines stable expressing T7 RNA polymerase (BHK-T7) were maintained in Dulbecco’s modified minimum essential medium (DMEM) (Gibco) containing 10% fetal bovine serum (FBS), 100 U/mL penicillin, and 100 μg/mL streptomycin. FCV strain 2280 and F9 were purchased from ATCC and propagated in CRFK cells.

### 2.2. Plasmid Construction

A reporter plasmid expressing firefly luciferase under the control of the feline IFN-β promoter (pIFN + 33-Luc) and plasmids expressing each viral protein of FCV strain 2280, named pFlag-p5.6, pFlag-p30, pFlag-p32, pFlag-p39, pFlag-Vpg, pFlag-PP, pFlag-VP1 and pFlag-VP2, were described previously [16]. Type II promoters, such as CMV, SV40, HSV-TK and T7 promoter, were cloned into the pGL3 vector using *Kpn*I and *Bgl*II restriction sites, resulting in the construction of the recombinant plasmids pCMV-Luc, pSV40-Luc, pHSV-TK-Luc and pT7-Luc. EGFP expression in the pEGFP-C1 vector (Clontech, Mountain View, CA, USA) is driven by the CMV promoter.

To screen the functional domain of 2280 PP that inhibits gene expression, N-terminal truncations of PP were constructed into the vector p3×Flag-CMV-10 between the *Kpn*I and *BamH*I restriction sites. FCV F9 PP_N-263_ was cloned into p3×Flag-CMV-10 between the *Kpn*I and *BamH*I restriction sites. The 3C-L protein genes of human norovirus Hu/NLV/GII/MD145-12/1987/US and Hu/GII.4/Sydney/NSW0514/2012/AU were chemically synthesized according to the sequences available in GenBank and then were cloned into the p3×Flag-CMV-10 vector between the *Kpn*I and *BamH*I restriction sites. The plasmids obtained were named pFlag-3CLP-87 and pFlag-3CLP-12. Site-specific mutation in this study was performed using a fusion PCR method.

### 2.3. Luciferase Assay

CRFK cells or BHK cells were transfected with types of luciferase gene reporter plasmids using Lipofectamine 2000 (Invitrogen, Grand Island, NY, USA), and the luciferase activity was measured according to the Dual-Luciferase Reporter Assay System protocol (Promega, Madison, WI, USA). To test luciferase activity driven by the feline IFN-β promoter, Sendai virus (SeV) with 100 hemagglutination (HA) units was inoculated as an interferon pathway activator, and the luciferase assay was performed 8–10 h post-inoculation. Individual luciferase expression from each sample was normalized to the level of the positive control transfected only with luciferase gene reporter plasmids, which was set as 1.

### 2.4. Capped RNA in Vitro Transcription

The pT7-Luc plasmid was linearized. After purification, the linearized plasmid was added to a transcription reaction containing T7 RNA polymerase and Ribo m7G cap analog according to the instructions of the Riboprobe^®^ in vitro Transcription Systems (Promega), and the reaction was incubated at 37 °C for 2 h. Then, RNase-Free DNase at a concentration of 1 U/μg of template DNA was added to the reaction to remove the DNA template, and the reaction was incubated for 15 min at 37 °C, followed by purification of the RNA with an RNeasy Mini kit (QIAGEN, Valencia, VA, USA).

### 2.5. Quantititive PCR Assay

Total RNA was prepared with an RNeasy Mini Kit (QIAGEN). Following treatment with RNase-Free Dnase at 37 °C for 1 h, RNA was purified again. cDNA was prepared according to the AMV-reverse transcription kit (Takara, Dalian, China). Real-time quantitative PCR targeting the luciferase gene was carried out using an Aligent Mx3005P according to the manufacturer’s instructions. The relative mRNA expression levels were calculated by the 2^−∆∆CT^ method using GADPH as an internal control for normalization. The following primers were used: Luc-forward 5′-GATTTCAGTCGATGTACACGT-3′, Luc-reverse 5′-AGACCAGTAGATCCAGAGGAG-3′; GAPDH-forward 5′-TGACCACAGTCCATGCCATC-3′, GAPDH-reverse: 5′-GCCAGTGAGCTTCCCGTTCA-3′.

### 2.6. Western Blot Analysis

The cell monolayers were washed once gently with PBS and lysised by 1× RIPA Lysis buffer containing 1% PMSF (Beyotime, Beijing, China). The lysates were cleared by centrifugation at 10,000 g for 5 m at 4 °C. The amount of total protein content was determined with a BCA protein assay kit (Beyotime). Total of 25 μg prepared sample was separated by SDS-PAGE and transferred onto nitrocellulose membranes by the G2 Fast Blotter (Thermo, Waltham, MA, USA). The membranes were blocked with 5% skim milk for 1 h at 37 °C, then incubated at room temperature for 2 h with specific rabbit anti-IFN-β antibody (ab140211, Abcam, Cambridge, MA, USA), rabbit anti-Flag antibody (ab1162, Abcam), rabbit anti-GADPH antibody (ab22555, Abcam), rabbit anti-eIF4GI (ab2609, Abcam) and rabbit anti-eIF4GII (ab83330, Abcam). After washing membranes using TBST buffer, the membranes were incubated at 37 °C for 1 h with IRDye 800DX conjugated anti-rabbit IgG (1:8000; Rockland Immunochemicals, Limerick, PA, USA) diluted in TBST buffer as secondary antibody. The membranes were washed three times in TBST, then visualized and analyzed with an Odyssey infrared imaging system (LI-COR Biosciences, Lincoln, NE, USA).

### 2.7. Statistics

The data are presented as the means ± standard deviation (SD). Statistical significance was determined using unpaired *t* tests and a one-way ANOVA in Prism 5.0 software (GraphPad Software, La Jolla, CA, USA). For all tests, a value of *p* < 0.05 was considered to indicate a significant difference.

## 3. Results

### 3.1. FCV Strain 2280 PP Suppresses Endogenous and Exogenous Gene Expression

**Figure 1:**
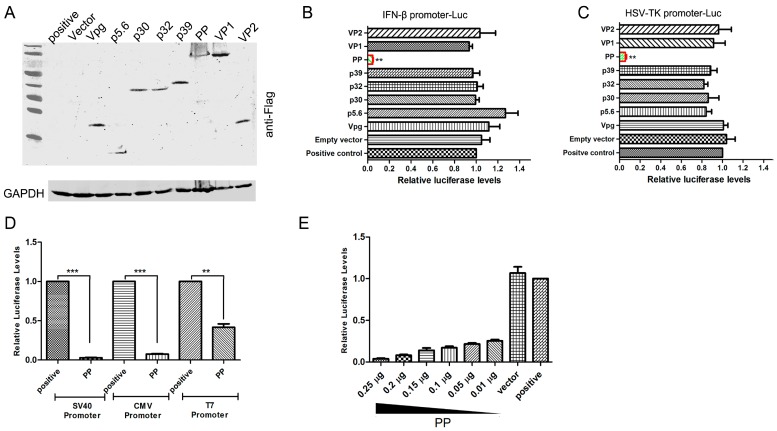

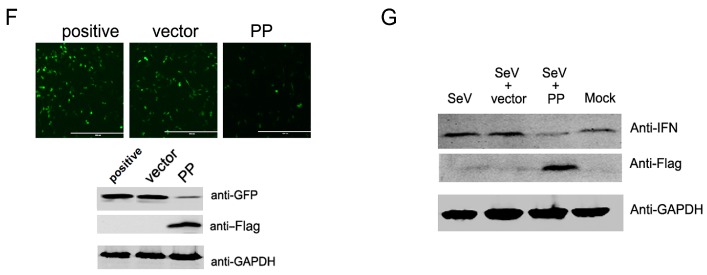
Detection of suppression of endogenous and exogenous promoters by the Feline Calicivirus (FCV) 2280 PP. (**A**) Identification of plasmids expressing viral proteins by Western blot; (**B**,**C**) Crandell-Reese feline kidney (CRFK) cells were transfected with 0.25 μg of pFlag-p5.6, pFlag-p30, pFlag-p32, pFlag-p39, pFlag-Vpg, pFlag-PP, pFlag-VP1, pFlag-VP2 or empty vector together with 0.25 μg pIFN + 33-Luc (**B**) or pHSV-TK-Luc (**C**) plasmids; At 24 h post-transfection, Sendai virus (SeV) (100 HA units) was inoculated into the pIFN+33-Luc transfection groups, and the luciferase assay was performed at 10 h post-inoculation. For the pHSV-TK-Luc transfection groups, the luciferase assay was performed directly at 24 h post-transfection; (**D**) The luciferase activities in CRFK cells transfected with 0.25 μg of pSV40-Luc or pCMV-Luc together with 0.25 μg of pFlag-PP were determined at 24 h post transfection. Evaluation of the effect of PP on T7 promoter activity was performed using BHK-T7 cells; (**E**) CRFK cells were transfected with 0.25 μg of pHSV-TK-Luc together with different doses of pFlag-PP or empty vector, and luciferase activities were determined at 24 h post-transfection. The positive controls (**B**–**E**) were only transfected with 0.25 μg of plasmids encoding reporter gene luciferase; (**F**) CRFK cells were transfected with 0.5 μg of pEGFP-C1 or 0.5 μg of pEGFP-C1 and 0.5 μg of pFlag-PP or empty vector. Fluorescence microscopy and Western blot evaluation of eGFP expression were performed at 24 h post-transfection; (**G**) CRFK cells were transfected with 1 μg of pFlag-PP or empty vector. SeV (500 HA units) was inoculated into the transfection groups or non-transfection groups at 24 h post-transfection. The mock group was treated with no transfection and no SeV inoculation. The expression of IFN-β was determined by Western blotting using an anti-IFN-β antibody at 12 h post-inoculation. Three independent experiments were performed and produced consistent results. The data represented the result of one experiment and were presented as means ± SD. **, *p* < 0.01; ***, *p* < 0.001.

To identify which FCV viral proteins could inhibit the expression of luciferase under the control of an endogenous promoter, the feline IFN-β promoter, CRFK cells were cotransfected with the pIFN-β-Luc plasmid and each viral gene expression plasmid (Figure 1A). As an interferon pathway stimulator, SeV was inoculated 24 h post-transfection, and a luciferase activity was performed 10 h post-inoculation. The results showed that the presence of p5.6, p30, p32, p39, VPg, VP1 and VP2 did not affect the luciferase activity, but PP expression led to a significant 90% reduction in luciferase activity (Figure 1B and Figure S1A). A constitutively exogenous promoter, the HSV-TK promoter, was also inhibited by the expression of PP (Figure 1C and Figure S1B). The poliovirus-encoded 3C protease has been shown to shut off cellular RNA polymerase II-mediated transcription [17]. To investigate whether PP-mediated shut-off of host gene expression resulted from the inhibition of the host RNA polymerase II (RNAP II) promoter, the activation of other RNAPII promoters, such as SV40, CMV and T7, was tested. As shown in Figure 1D and Figure S1C, PP reduced the luciferase gene expression driven by the SV40, CMV and T7 promoters. We also found that the PP-mediated shut-off of reporter gene expression was in a dose-dependent manner (Figure 1E and Figure S1D), and the transfection of 10 ng contributed to an 80% decrease, indicating a high inhibition efficiency.

To eliminate the possibility that PP only targeted luciferase reporter gene expression specifically, the eGFP gene was used to replace the luciferase gene under the control of the CMV promoter, and the expression of the endogenous IFN-β gene was evaluated in the presence of PP. Similar to the previous results, PP also inhibited the expression of eGFP (Figure 1F) and IFN-β induced by SeV infection (Figure 1G). These results suggested that the FCV strain 2280 PP suppresses endogenous and exogenous gene expression driven by RNAP II promoters.

### 3.2. The N-Terminal Domain of PP Is Responsible for the Suppression of Host Gene Expression

**Figure 2:**
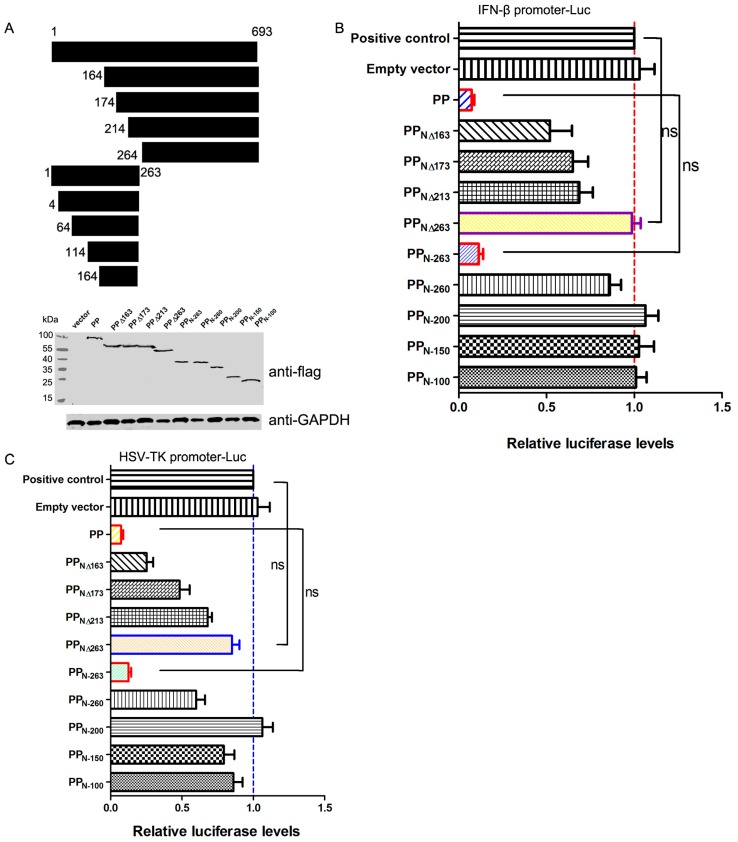
Screening the key domain of proteinase-polymerase (PP) required for the suppression of host gene expression. (**A**) Schematic diagram of the truncated PP constructs and the expression of each truncated mutant evaluated by Western blot using anti-Flag antibody; (**B**,**C**) CRFK cells were transfected with 0.25 μg of pFlag-PP and each truncated construct or empty vector together with 0.25 μg of the pIFN+33-Luc (**B**) or pHSV-TK-Luc (**C**) plasmids; At 24 h post-transfection, SeV (100 HA units) was inoculated into the pIFN+33-Luc transfection groups, and the luciferase assay was performed at 10 h post-inoculation. For the pHSV-TK-Luc transfection groups, the luciferase assay was performed directly at 24 h post-transfection. The positive controls were only transfected with 0.25 μg of pIFN+33-Luc (**B**) or pHSV-TK-Luc (**C**) Three independent experiments were performed and produced consistent results. The data represented the result of one experiment and were presented as means ± SD. ns, non-significant.

FCV PP is a bifunctional proteinase-polymerase protein [9]. To elucidate the key domain of PP for its shut-off activity, PP was truncated gradually at the N terminus, and the expression of each truncated PP was identified by Western blotting using anti-Flag antibody, as shown in Figure 2A. The effect of each truncated PP on luciferase gene expression under the control of the feline IFN-β promoter (Figure 2B) and the HSV-TK promoter (Figure 2C) was determined using a luciferase assay. As shown in Figure 2B,C, deletion of 163 aa, 173 aa and 213 aa from the N-terminus reduced the ability of PP to inhibit reporter gene expression, but did not lead to the complete loss of PP function. Deletion of the N-terminal 263 aa completely disrupted the function of PP (Figure 2B,C), and expression of the N-terminal 263 aa of PP (PP_N-263_) produced a similar inhibitory effect as that obtained with full-length PP (Figure 2B,C), suggesting that this region was important for the suppression of host gene expression. Additionally, we found that PP_N-263_-mediated shut-off depended on the complete N-terminus, and deletion of 3 aa at the N-terminus (PP_N-260_) only led to 14%–40% inhibitory ability (Figure 2B,C). Thus, the N-terminal 263 amino acids of FCV 2280 PP determine its shut-off activity.

### 3.3. PP Val27, Ala96 and Ala98 Are Key Sites for N-Terminus-Mediated Shut-Off Activity

**Figure 3:**
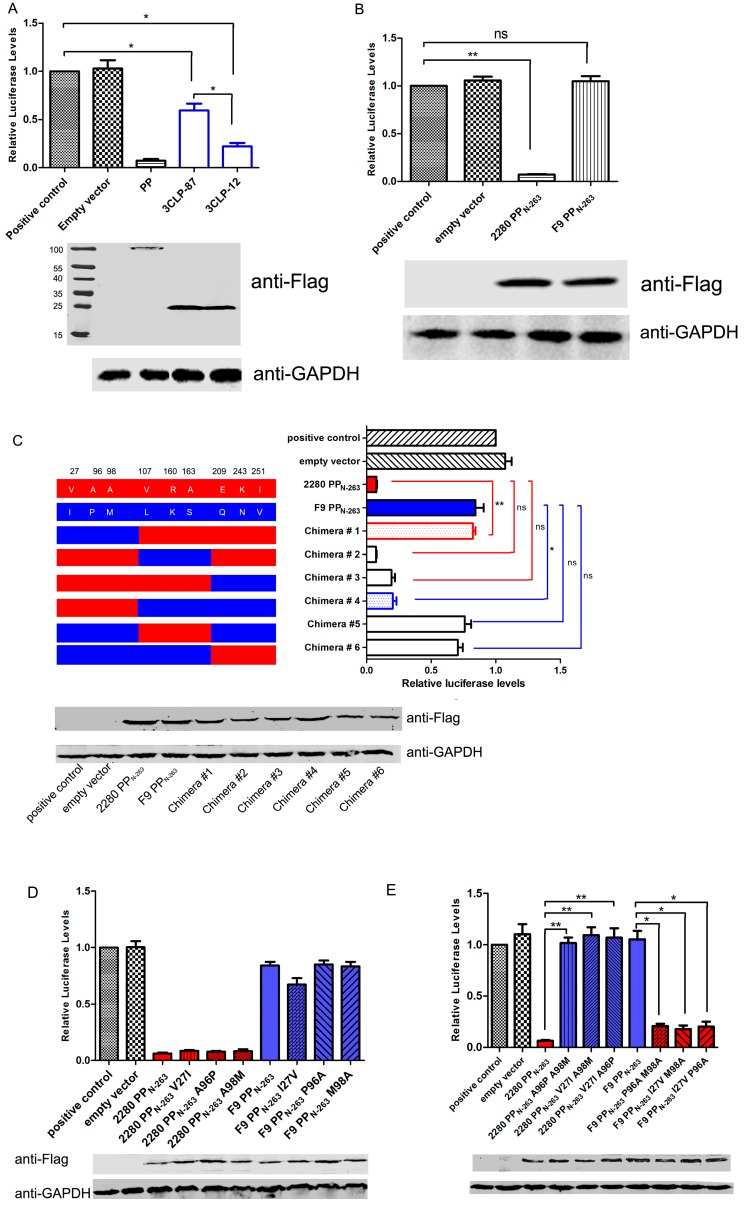
Identification of the key sites of PP for inhibiting host gene expression. (**A**,**B**) CRFK cells were transfected with 0.25 μg of pIFN+33-Luc together with 0.25 μg of pFlag-3CLP-87 and pFlag-3CLP-12 (**A**); 2280 PP_N-263_ and F9 PP_N-263_ (**B**) or empty vector. At 24 h post-transfection, SeV was inoculated into the cells for 10 h, and then cell lysates were subjected to luciferase assay and Western blot analysis using anti-Flag antibody; (**C–E**) Luciferase expression from pIFN+33-Luc in cells cotransfected with the indicated chimeric PP genes (**C**) or single (**D**) or double mutants (**E**) of PP in the p3×Flag-CMV vector. The expression of each construct was determined by Western blotting using an anti-Flag antibody. Anti-GAPDH was used as a loading control. Three independent experiments were performed and produced consistent results. The data represented the result of one experiment and were presented as means ± SD. *, *p* < 0.05; **, *p* < 0.01; ns, non-significant.

Amino acid 163 of PP may be a potential boundary between its proteinase domain and its polymerase domain [18], and the proteinase activity of PP depends on its N-terminus. Because previous results have shown that the proteinase domain of PP is associated with its shut-off activity, we investigated the ability of norovirus 3C-like protein (3CLP) from the GII.4-1987 and -2012 isolates (3CLP-87 and 3CLP-12, respectively) to interrupt host gene expression. The result showed that 3CLP from both isolates was able to significantly interfere with the luciferase gene expression driven by the IFN-β promoter, but 3CLP-12 had a stronger inhibitory activity than 3CLP-12 (Figure 3A). Furthermore, we compared the shut-off activity of the N-terminal 263 aa from strains 2280 (2280 PP_N-263_) and F9 (F9 PP_N-263_). The expression of F9 PP_N-263_ could not inhibit the luciferase gene expression driven by the IFN-β promoter (Figure 3B) and other promoters. There are nine amino acid differences in this domain between F9 and 2280 PP (Figure 3C left). To identify the residues required for the shut-off activity, six PP chimeras composed of F9 and 2280 PP_N-263_ (Figure 3C left) were created and cloned into the p3×Flag-CMV-10 vector, and expression in CRFK cells was determined by Western blotting using anti-Flag antibody (Figure 3C down). The effect of each PP chimera on host gene expression was evaluated by examining the luciferase gene expression driven by the IFN-β promoter using a luciferase assay (Figure 3C right). Comparison of the levels of luciferase revealed that residues within the N-terminal 100 amino acids of PP are responsible for its inhibitory activity. In the background of 2280 PP-N263, the expression of chimera #1 significantly attenuated the shut-off activity of 2280 PP_N-263_ (Figure 3C right). In the background of F9 PP_N-263_, the expression of chimera #4 significantly increased the shut-off activity of F9 PP_N-263_ (Figure 3C right).

Next, single mutations were created in PP_N-263_. As shown in Figure 3D, single site mutations at residues 27, 96 and 98 did not affect the shut-off activity of 2280 and F9 PP_N-263_. To further examine the roles of these sites, double site mutations were created in 2280 and F9 PP_N-263_. We found that mutation of any two sites resulted in the elimination of the shut-off activity in the 2280 PP_N-263_ and the presence of the shut-off activity in the F9 PP_N-263_ (Figure 3E). The data showed that residues Val27, Ala96 and Ala98 were key sites for the shut-off activity of PP_N-263_.

### 3.4. FCV 2280 PP Inhibits Host Gene Transcription

**Figure 4:**
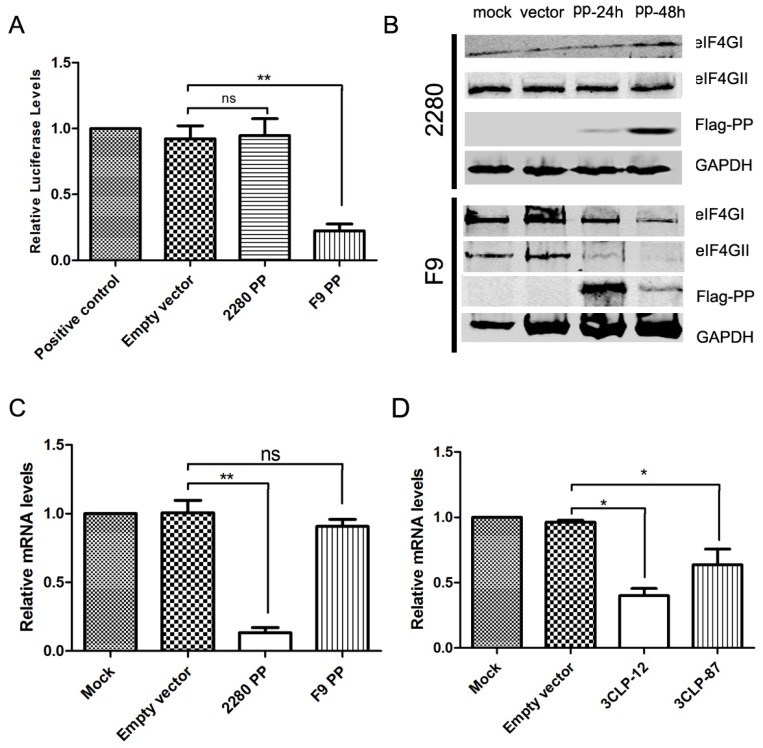
Analysis of PP inhibition of host gene transcription. (**A**) Effect of PP on mRNA translation. CRFK cells were transfected with 0.25 μg of capped luciferase mRNA together with 0.25 μg of pFlag-2280 PP and pFlag-F9 PP or empty vector. No transfection or transfection with capped luciferase mRNA only were used as the negative control and positive control, respectively; (**B**) Effect of PP on the expression of the translation initiation factors eIF4GI and eIF4GII. CRFK cells were transfected with 1 μg of pFlag-2280 PP and pFlag-F9 PP or empty vector for 24 and 48 h, and the expression of eIF4GI and eIF4GII was determined by Western blotting using anti-eIF4GI and -eIF4GII antibodies; (**C**,**D**) Effect of PP and 3CLP on gene transcription. CRFK cells were transfected with 0.25 μg of pIFN+33-Luc together with 0.25 μg of pFlag-2280 PP and pFlag-F9 PP (**C**) and pFlag-3CLP-12 and -3CLP-87 (**D**) or empty vector. Transfection with pIFN+33-Luc only was used as a positive control. The levels of luciferase mRNA were determined by qPCR. Three independent experiments were performed and produced consistent results. The data represented the result of one experiment and were presented as means ± SD. *, *p* < 0.05; **, *p* < 0.01; ns, non-significant.

To identify whether host cell translation was inhibited by FCV PP, the vector pT7-Luc, expressing luciferase under the control of the T7 promoter, was constructed, and a capped luciferase RNA was produced using a T7 in vitro transcription kit. CRFK cells were transfected with pFlag-PP for 24 h and then transfected with the capped luciferase RNA. The luciferase assay was performed 12 h post-transfection. As shown in Figure 4A, F9 PP significantly inhibited the RNA translation, but 2280 PP did not affect the RNA translation. During viral infection, some proteins, such as the translation initiation factors eIF4GI and eIF4GII, are cleaved by viral proteins [11]. A previous report demonstrated that infection of FCV strain F9 led to the inhibition of cellular protein synthesis, which is accompanied by the cleavage of the eukaryotic translation initiation factors eIF4GI and eIF4GII [11]. However, this report did not suggest which viral proteins were responsible for it. We found that expression of F9 PP decreased the level of the eIF4GII (Figure 4B), but expression of 2280 PP did not affect the level of either of both proteins in cells (Figure 4B).

To identify whether host cell transcription was inhibited by 2280 PP, CRFK cells were co-transfected with pFlag-PP and pIFN+33-Luc. At 24 h post-transfection, SeV was inoculated into the cells to induce the expression of the luciferase gene, and the levels of luciferase mRNA were detected by real-time PCR at 10 h post-inoculation. Expression of F9 PP did not affect the production of luciferase mRNA, but expression of 2280 PP contributed to a 98% reduction (Figure 4C). We also evaluated the effect of 3CLP-12 and -87 on the production of luciferase mRNA using similar methods, and we found that both 3CLP-12 and -87 decreased the production of luciferase mRNA with different levels (Figure 4D).

## 4. Discussion

FCV has developed diverse strategies to maximize viral protein synthesis and shut off host protein synthesis [19]. In this study, we found that FCV strain 2280 PP inhibited the exogenous luciferase gene expression driven by the endogenous feline IFN-β promoter as well as the exogenous promoters HSV-TK, SV40, CMV and T7 and also inhibited the endogenous feline IFN-β expression induced by SeV. The N-terminal 263 aa of PP are responsible for the suppression of host gene expression. A series of comparison experiments further indicated that residues Val27, Ala96 and Ala98 were the key sites for the shut-off activity of PP. A preliminary investigation into the mechanism revealed that the suppression of reporter gene transcription induced by PP contributed to the shut-off activity.

Viruses regulate host cell transcription/translation machinery for the preferential production of viral proteins. Many viruses have been reported to shut off host protein synthesis, such as influenza virus [20], Sindbis virus [21], Potato virus A [22] and poliovirus [23]. The influenza virus PA protein is responsible for the suppression of host protein synthesis [24]. Shut-off of RNA polymerase II transcription by poliovirus involves the 3CPro protease-mediated cleavage of the TATA-binding protein [23]. Although a previous study reported that eIF4G was specifically cleaved into smaller proteins during FCV strain F9 infection, here, we detected intact eIF4GI and II in cells transfected with the FCV strain 2280 PP, which revealed that PP does not directly proteolyze eIF4G. In this study, PP was shown to repress cellular gene expression by modulating host gene transcription. Viruses must rely on host cell machinery for efficient replication and production of progeny virions. In the process of virus evolution, virus aquires abitity to hijacks cellular machinery for its own benefit. Inhibiting host gene transcription by PP would also suppress host antiviral response, which could be a major factor for efficient viral spread and pathogenicity. So, FCV PP may play an important role in promoting viral replication and inhibiting host antiviral response.

FCV is a positive-strand RNA virus with high evolutionary rates; the variable regions of the capsid protein have been estimated to undergo 1.3 × 10^−2^ to 2.6 × 10^−2^ substitutions per nucleotide per year [25]. The PP proteins of FCV 2280 and F9 share 94% amino acid identity. The in vitro growth kinetics of strain 2280 were faster than those of strain F9, and challenge experiments in cats showed that strain 2280 infection exhibited more virulence than strain F9 [26]. Viral protease and polymerase activity may contribute to these differences. Here, the PP protein N-termini of the two strains had different functions, although shut-off of host-cell protein synthesis was also observed in FCV F9 infection. We found that the residues Val27, Ala96 and Ala98 were key sites for the shut-off activity of the PP N-terminus. It remains to be determined whether these three sites affect FCV in vitro growth and in vivo virulence. We analyzed the variation of the three residues from a total of 48 FCV strains and found that five strains (GI number: 513038406; 49458053; 669081955; 98986307; 49458065) contain the same amino acid residues at the three positions of PP as strain 2280, indicating that this characteristic is not specific for only strain 2280 or F9.

## 5. Conclusions

In this study, we found that the suppression of host gene expression by strain 2280 PP was determined by N-terminal 263 aa of PP. Further, we demonstrated that Val27, Ala96 and Ala98 were key sites for the inhibition of host gene expression by strain 2280 PP. Our results provide new insight into the mechanism of the FCV-mediated inhibition of host gene expression.

## Supplementary Materials

Supplementary materials can be found at http://www.mdpi.com/1999-4915/8/7/199/s1.
