# Supplementary material for: N-Terminal Domain of Feline Calicivirus (FCV) Proteinase-Polymerase Contributes to the Inhibition of Host Cell Transcription
Source: Viruses. 2016 Jul 20;8(7):199. doi: 10.3390/v8070199 (PMC4974534; doi:10.3390/v8070199)
Supplement: Supplementary file 1 [file viruses-08-00199-s001.pdf]

Viruses **2016**, *8*, 199; doi:10.3390/v8070199

Supplementary Materials: Article N-Terminal Domain of Feline Calicivirus (FCV) Proteinase-Polymerase Contributes to the Inhibition of Host Cell Transcription

Hongxia Wu, Shaopo Zu, Xue Sun, Yongxiang Liu, Jin Tian and Liandong Qu

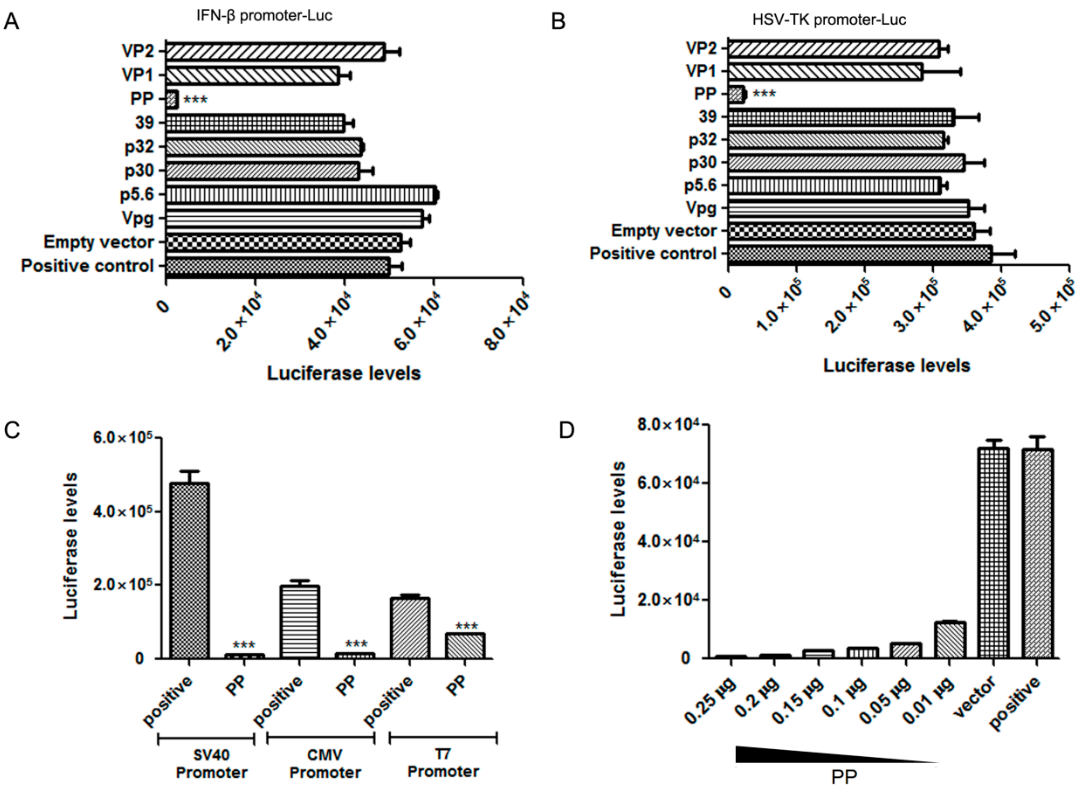

Figure S1. Analyses of absolute luciferase activities under control of endogenous and exogenous promoters in presense of each FCV 2280 protein. The methods in (**A**–**D**) refer to the protocol in Figure 1B–E. The data represent the absolute luciferase activities. Three independent experiments were performed and produced consistent results. The data represented the result of one experiment and were presented as means ± SD. ***, $p < 0.001$.

© 2016 by the authors. Submitted for possible open access publication under the terms and conditions of the Creative Commons Attribution (CC-BY) license (http://creativecommons.org/licenses/by/4.0/).
